# Supplementary material for: The reporting quality and spin of randomized controlled trials of endometriosis pain: Methodological study based on CONSORT extension on abstracts
Source: PLoS One. 2024 May 2;19(5):e0302108. doi: 10.1371/journal.pone.0302108 (PMC11065215; doi:10.1371/journal.pone.0302108)
Supplement: S1 File — (DOCX) [file pone.0302108.s002.docx]

**Appendix 1: The article metrics and characteristics of journals**

| **ID** | **year** | **Journal metrics** | | | | | | | **Article metrics** | | | | |
| --- | --- | --- | --- | --- | --- | --- | --- | --- | --- | --- | --- | --- | --- |
|  |  | **Journal name** | **indexing** | **Subject Category** | **Impact Factor** | **IF Quartile** | **Cite Score** | **H-Index** | **Abstract word count** | **authors** | **citations** | **FWCI ^a^** | **geographic location** |
| 1 | 2010  (22) | Human Reproduction | ISI, Scopus, PubMed, Embase | \| 5% \| Rehabilitation  Physical Therapy \| \| --- \| --- \|   Rehabilitation  Physical Therapy | [6.100](http://gateway.isiknowledge.com/gateway/Gateway.cgi?GWVersion=2&SrcAuth=JCR&SrcApp=JCR&DestApp=JCR&KeyRecord=0268-1161) | Q1 | [10.50](https://www.scopus.com/sourceid/27505) | [245](https://www.scimagojr.com/journalsearch.php?q=27505&tip=sid) | 251 | 5 | 274 | 7.67 | Europe |
| 2 | 2011  (23) | European Journal of Obstetrics & Gynecology and Reproductive Biology | ISI, Scopus, PubMed, Embase | Obstetrics & Gynecology  Nursing and Midwifery | [2.600](http://gateway.isiknowledge.com/gateway/Gateway.cgi?GWVersion=2&SrcAuth=JCR&SrcApp=JCR&DestApp=JCR&KeyRecord=0301-2115) | Q2 | [4.00](https://www.scopus.com/sourceid/12681) | [111](https://www.scimagojr.com/journalsearch.php?q=12681&tip=sid) | 239 | 7 | 61 | 1.98 | Europe |
| 3 | 2011  (24) | Reproductive Biology and Endocrinology | ISI, Scopus, PubMed, Embase, DOAJ | Biology  Endocrinology & Metabolism  Obstetrics & Gynecology  Nursing and Midwifery | [4.400](http://gateway.isiknowledge.com/gateway/Gateway.cgi?GWVersion=2&SrcAuth=JCR&SrcApp=JCR&DestApp=JCR&KeyRecord=1477-7827) | Q1 | [6.70](https://www.scopus.com/sourceid/26698) | [102](https://www.scimagojr.com/journalsearch.php?q=26698&tip=sid) | 318 | 4 | 57 | 1.09 | Italy |
| 4 | 2011  (25) | European Journal of Obstetrics and Gynecology and Reproductive Biology | ISI, Scopus, PubMed, Embase | Obstetrics & Gynecology  Nursing and Midwifery | [2.600](http://gateway.isiknowledge.com/gateway/Gateway.cgi?GWVersion=2&SrcAuth=JCR&SrcApp=JCR&DestApp=JCR&KeyRecord=0301-2115) | Q3 | [4.00](https://www.scopus.com/sourceid/12681) | [111](https://www.scimagojr.com/journalsearch.php?q=12681&tip=sid) | 328 | 4 | 51 | 1.85 | Japan |
| 5 | 2011  (26) | Zhongguo zhen jiu = Chinese acupuncture & moxibustion | [PubMed](https://www.ncbi.nlm.nih.gov/nlmcatalog/?term=0255-2930) | -- | -- | -- | -- | -- | 383 | 3 | 9 | 0.45 | China |
| 6 | 2012  (27) | Iranian Journal of Reproductive Medicine | [PubMed](https://www.ncbi.nlm.nih.gov/nlmcatalog/?term=1680-6433),  [DOAJ](https://doaj.org/toc/1680-6433) | Obstetrics & Gynecology  Nursing and Midwifery | -- | -- | -- | -- | 294 | 4 | 7 | 0.53 | Iran |
| 7 | 2012  (28) | Archives of Gynecology and Obstetrics | [ISI](http://mjl.clarivate.com/cgi-bin/jrnlst/jlresults.cgi?PC=MASTER&ISSN=0932-0067), [Scopus](https://www.scopus.com/sourceid/30082), [PubMed](https://www.ncbi.nlm.nih.gov/nlmcatalog/?term=0932-0067), [Embase](https://www.embase.com/search?sb=y&search_query='09320067':is) | General Medicine  Obstetrics & Gynecology | [2.600](http://gateway.isiknowledge.com/gateway/Gateway.cgi?GWVersion=2&SrcAuth=JCR&SrcApp=JCR&DestApp=JCR&KeyRecord=0932-0067) | Q3 | [4.50](https://www.scopus.com/sourceid/30082) | [79](https://www.scimagojr.com/journalsearch.php?q=30082&tip=sid) | 237 | 8 | 150 | 7.79 | Germany, Italy and Ukraine |
| 8 | 2012  (29) | Obstetrics and Gynecology | [ISI](http://mjl.clarivate.com/cgi-bin/jrnlst/jlresults.cgi?PC=MASTER&ISSN=0029-7844), [Scopus](https://www.scopus.com/sourceid/28089), [PubMed](https://www.ncbi.nlm.nih.gov/nlmcatalog/?term=0029-7844), [Embase](https://www.embase.com/search?sb=y&search_query='00297844':is) | \| 5% \| Obstetrics & Gynecology \| \| --- \| --- \| | [7.200](http://gateway.isiknowledge.com/gateway/Gateway.cgi?GWVersion=2&SrcAuth=JCR&SrcApp=JCR&DestApp=JCR&KeyRecord=0029-7844) | Q1 | [9.80](https://www.scopus.com/sourceid/28089) | [245](https://www.scimagojr.com/journalsearch.php?q=28089&tip=sid) | 287 | 6 | 90 | 4.04 | Thailand |
| 9 | 2013  (30) | Medical Science Monitor | [ISI](http://mjl.clarivate.com/cgi-bin/jrnlst/jlresults.cgi?PC=MASTER&ISSN=1234-1010), [Scopus](https://www.scopus.com/sourceid/18344), [PubMed](https://www.ncbi.nlm.nih.gov/nlmcatalog/?term=1234-1010), [Embase](https://www.embase.com/search?sb=y&search_query='12341010':is) | General Medicine | [3.100](http://gateway.isiknowledge.com/gateway/Gateway.cgi?GWVersion=2&SrcAuth=JCR&SrcApp=JCR&DestApp=JCR&KeyRecord=1234-1010) | Q3 | [6.60](https://www.scopus.com/sourceid/18344) | [97](https://www.scimagojr.com/journalsearch.php?q=18344&tip=sid) | 99 | 15 | 38 | 2.88 | Iran |
| 10 | 2013  (31) | Journal of Obstetrics and Gynaecology Research | [ISI](http://mjl.clarivate.com/cgi-bin/jrnlst/jlresults.cgi?PC=MASTER&ISSN=1341-8076), [Scopus](https://www.scopus.com/sourceid/27608), [PubMed](https://www.ncbi.nlm.nih.gov/nlmcatalog/?term=1341-8076), [Embase](https://www.embase.com/search?sb=y&search_query='13418076':is) | Obstetrics & Gynecology | [1.600](http://gateway.isiknowledge.com/gateway/Gateway.cgi?GWVersion=2&SrcAuth=JCR&SrcApp=JCR&DestApp=JCR&KeyRecord=1341-8076) | Q4 | [2.80](https://www.scopus.com/sourceid/27608) | [59](https://www.scimagojr.com/journalsearch.php?q=27608&tip=sid) | 245 | 5 | 45 | 2.01 | Italy |
| 11 | 2013  (32) | [Translational Research](http://www.journals.elsevier.com/translational-research-the-journal-of-laboratory-and-clinical-medicine/) | [ISI](http://mjl.clarivate.com/cgi-bin/jrnlst/jlresults.cgi?PC=MASTER&ISSN=1878-1810), [Scopus](https://www.scopus.com/sourceid/4700152753), [PubMed](https://www.ncbi.nlm.nih.gov/nlmcatalog/?term=1878-1810), [Embase](https://www.embase.com/search?sb=y&search_query='18781810':is) | Occupational Medicine  Environmental Health  Occupational health  General Medicine  Biochemistery | [7.800](http://gateway.isiknowledge.com/gateway/Gateway.cgi?GWVersion=2&SrcAuth=JCR&SrcApp=JCR&DestApp=JCR&KeyRecord=1878-1810) | Q1 | [15.00](https://www.scopus.com/sourceid/4700152753) | [111](https://www.scimagojr.com/journalsearch.php?q=4700152753&tip=sid) | 255 | 5 | 97 | 2.43 | United States |
| 12 | 2014  (33) | Medical Journal of the Islamic Republic of Iran | [Scopus](https://www.scopus.com/sourceid/17840), [PubMed](https://www.ncbi.nlm.nih.gov/nlmcatalog/?term=1016-1430) | General Medicine | -- | -- | [2.10](https://www.scopus.com/sourceid/17840) | [31](https://www.scimagojr.com/journalsearch.php?q=17840&tip=sid) | 138 | 4 | 7 | 0.78 | Iran |
| 13 | 2014  (34) | European Journal of Obstetrics and Gynecology and Reproductive Biology | [ISI](http://mjl.clarivate.com/cgi-bin/jrnlst/jlresults.cgi?PC=MASTER&ISSN=0301-2115), [Scopus](https://www.scopus.com/sourceid/12681), [PubMed](https://www.ncbi.nlm.nih.gov/nlmcatalog/?term=0301-2115),  [Embase](https://www.embase.com/search?sb=y&search_query=%2703012115%27:is) | Obstetrics & Gynecology  Nursing and Midwifery | [2.600](http://gateway.isiknowledge.com/gateway/Gateway.cgi?GWVersion=2&SrcAuth=JCR&SrcApp=JCR&DestApp=JCR&KeyRecord=0301-2115) | Q3 | [4.00](https://www.scopus.com/sourceid/12681) | [111](https://www.scimagojr.com/journalsearch.php?q=12681&tip=sid) | 201 | 5 | 59 | 2.76 | Italy |
| 14 | 2014  (35) | European Journal of Obstetrics and Gynecology and Reproductive Biology | [ISI](http://mjl.clarivate.com/cgi-bin/jrnlst/jlresults.cgi?PC=MASTER&ISSN=0301-2115), [Scopus](https://www.scopus.com/sourceid/12681), [PubMed](https://www.ncbi.nlm.nih.gov/nlmcatalog/?term=0301-2115),  [Embase](https://www.embase.com/search?sb=y&search_query=%2703012115%27:is) | Obstetrics & Gynecology  Nursing and Midwifery | [2.600](http://gateway.isiknowledge.com/gateway/Gateway.cgi?GWVersion=2&SrcAuth=JCR&SrcApp=JCR&DestApp=JCR&KeyRecord=0301-2115) | Q3 | [4.00](https://www.scopus.com/sourceid/12681) | [111](https://www.scimagojr.com/journalsearch.php?q=12681&tip=sid) | 303 | 6 | 10 | 0.65 | Austria |
| 15 | 2015  (36) | Journal of Minimally Invasive Gynecology | [ISI](http://mjl.clarivate.com/cgi-bin/jrnlst/jlresults.cgi?PC=MASTER&ISSN=1553-4650), [Scopus](https://www.scopus.com/sourceid/144915), [PubMed](https://www.ncbi.nlm.nih.gov/nlmcatalog/?term=1553-4650), [Embase](https://www.embase.com/search?sb=y&search_query='15534650':is) | Obstetrics & Gynecology | [4.100](http://gateway.isiknowledge.com/gateway/Gateway.cgi?GWVersion=2&SrcAuth=JCR&SrcApp=JCR&DestApp=JCR&KeyRecord=1553-4650) | Q1 | [4.40](https://www.scopus.com/sourceid/144915) | [88](https://www.scimagojr.com/journalsearch.php?q=144915&tip=sid) | 263 | 7 | 10 | 0.42 | United States |
| 16 | 2015  (37) | Acta Obstetricia et Gynecologica Scandinavica | [ISI](http://mjl.clarivate.com/cgi-bin/jrnlst/jlresults.cgi?PC=MASTER&ISSN=0001-6349), [Scopus](https://www.scopus.com/sourceid/30056), [PubMed](https://www.ncbi.nlm.nih.gov/nlmcatalog/?term=0001-6349), [Embase](https://www.embase.com/search?sb=y&search_query='00016349':is) | General Medicine  Obstetrics & Gynecology | [4.300](http://gateway.isiknowledge.com/gateway/Gateway.cgi?GWVersion=2&SrcAuth=JCR&SrcApp=JCR&DestApp=JCR&KeyRecord=0001-6349) | Q1 | [7.30](https://www.scopus.com/sourceid/30056) | [114](https://www.scimagojr.com/journalsearch.php?q=30056&tip=sid) | 221 | 8 | 42 | 3.43 | Italy |
| 17 | 2016  (38) | Medical Science Monitor | [ISI](http://mjl.clarivate.com/cgi-bin/jrnlst/jlresults.cgi?PC=MASTER&ISSN=1234-1010), [Scopus](https://www.scopus.com/sourceid/18344), [PubMed](https://www.ncbi.nlm.nih.gov/nlmcatalog/?term=1234-1010), [Embase](https://www.embase.com/search?sb=y&search_query='12341010':is) | General Medicine | [3.100](http://gateway.isiknowledge.com/gateway/Gateway.cgi?GWVersion=2&SrcAuth=JCR&SrcApp=JCR&DestApp=JCR&KeyRecord=1234-1010) | Q3 | [6.60](https://www.scopus.com/sourceid/18344) | [97](https://www.scimagojr.com/journalsearch.php?q=18344&tip=sid) | 220 | 5 | 40 | 1.70 | Iran |
| 18 | 2016  (39) | Obstetrics and Gynecology | [ISI](http://mjl.clarivate.com/cgi-bin/jrnlst/jlresults.cgi?PC=MASTER&ISSN=0029-7844), [Scopus](https://www.scopus.com/sourceid/28089), [PubMed](https://www.ncbi.nlm.nih.gov/nlmcatalog/?term=0029-7844), [Embase](https://www.embase.com/search?sb=y&search_query='00297844':is) | \| 5% \| Obstetrics & Gynecology \| \| --- \| --- \| | [7.200](http://gateway.isiknowledge.com/gateway/Gateway.cgi?GWVersion=2&SrcAuth=JCR&SrcApp=JCR&DestApp=JCR&KeyRecord=0029-7844) | Q1 | [9.80](https://www.scopus.com/sourceid/28089) | [245](https://www.scimagojr.com/journalsearch.php?q=28089&tip=sid) | 289 | 8 | 46 | 1.70 | Germany |
| 19 | 2017  (40) | Journal of the Endocrine Society | [ESCI (ISI)](http://mjl.clarivate.com/cgi-bin/jrnlst/jlresults.cgi?PC=MASTER&ISSN=2472-1972),  [Scopus](https://www.scopus.com/sourceid/21100941739),  [PubMed](https://www.ncbi.nlm.nih.gov/nlmcatalog/?term=2472-1972) |  | [4.100](http://gateway.isiknowledge.com/gateway/Gateway.cgi?GWVersion=2&SrcAuth=JCR&SrcApp=JCR&DestApp=JCR&KeyRecord=2472-1972) | -- | [6.40](https://www.scopus.com/sourceid/21100941739) | [34](https://www.scimagojr.com/journalsearch.php?q=21100941739&tip=sid) | 237 | 5 | 40 | 1.25 | Brazil |
| 20 | 2017  (41) | Fertility and Sterility | [ISI](http://mjl.clarivate.com/cgi-bin/jrnlst/jlresults.cgi?PC=MASTER&ISSN=0015-0282), [Scopus](https://www.scopus.com/sourceid/12705), [PubMed](https://www.ncbi.nlm.nih.gov/nlmcatalog/?term=0015-0282), [Embase](https://www.embase.com/search?sb=y&search_query='00150282':is) | \| 5% \| Obstetrics & Gynecology \| \| --- \| --- \| | [6.700](http://gateway.isiknowledge.com/gateway/Gateway.cgi?GWVersion=2&SrcAuth=JCR&SrcApp=JCR&DestApp=JCR&KeyRecord=0015-0282) | Q1 | [10.50](https://www.scopus.com/sourceid/12705) | [230](https://www.scimagojr.com/journalsearch.php?q=12705&tip=sid) | 268 | 6 | 53 | 3.16 | Japan |
| 21 | 2017  (42) | New England Journal of Medicine | [ISI](http://mjl.clarivate.com/cgi-bin/jrnlst/jlresults.cgi?PC=MASTER&ISSN=0028-4793), [Scopus](https://www.scopus.com/sourceid/15847), [PubMed](https://www.ncbi.nlm.nih.gov/nlmcatalog/?term=0028-4793), [Embase](https://www.embase.com/search?sb=y&search_query='00284793':is) | \| 1% \| General Medicine \| \| --- \| --- \| | [158.500](http://gateway.isiknowledge.com/gateway/Gateway.cgi?GWVersion=2&SrcAuth=JCR&SrcApp=JCR&DestApp=JCR&KeyRecord=0028-4793) | Q1 | [134.40](https://www.scopus.com/sourceid/15847) | [1,130](https://www.scimagojr.com/journalsearch.php?q=15847&tip=sid) | 429 | 21 | 321 | 21.33 | United States and Canada |
| 22 | 2018  (43) | Fertility and Sterility | [ISI](http://mjl.clarivate.com/cgi-bin/jrnlst/jlresults.cgi?PC=MASTER&ISSN=0301-2115), [Scopus](https://www.scopus.com/sourceid/12681), [PubMed](https://www.ncbi.nlm.nih.gov/nlmcatalog/?term=0301-2115),  [Embase](https://www.embase.com/search?sb=y&search_query=%2703012115%27:is) | \| 5% \| Obstetrics & Gynecology \| \| --- \| --- \| | [6.700](http://gateway.isiknowledge.com/gateway/Gateway.cgi?GWVersion=2&SrcAuth=JCR&SrcApp=JCR&DestApp=JCR&KeyRecord=0015-0282) | Q1 | [10.50](https://www.scopus.com/sourceid/12705) | [230](https://www.scimagojr.com/journalsearch.php?q=12705&tip=sid) | 258 | 5 | 58 | 3.50 | Brazil |
| 23 | 2018  (44) | European Journal of Obstetrics & Gynecology and Reproductive Biology | [ISI](http://mjl.clarivate.com/cgi-bin/jrnlst/jlresults.cgi?PC=MASTER&ISSN=0301-2115), [Scopus](https://www.scopus.com/sourceid/12681), [PubMed](https://www.ncbi.nlm.nih.gov/nlmcatalog/?term=0301-2115),  [Embase](https://www.embase.com/search?sb=y&search_query=%2703012115%27:is) | Obstetrics & Gynecology  Nursing and Midwifery | [2.600](http://gateway.isiknowledge.com/gateway/Gateway.cgi?GWVersion=2&SrcAuth=JCR&SrcApp=JCR&DestApp=JCR&KeyRecord=0301-2115) | Q2 | [4.00](https://www.scopus.com/sourceid/12681) | [111](https://www.scimagojr.com/journalsearch.php?q=12681&tip=sid) | 147 | 4 | 25 | 1.32 | Spain |
| 24 | 2018  (45) | European Journal of Contraception and Reproductive Health Care | [ISI](http://mjl.clarivate.com/cgi-bin/jrnlst/jlresults.cgi?PC=MASTER&ISSN=1362-5187), [Scopus](https://www.scopus.com/sourceid/24635), [PubMed](https://www.ncbi.nlm.nih.gov/nlmcatalog/?term=1362-5187), [Embase](https://www.embase.com/search?sb=y&search_query='13625187':is) | Obstetrics & Gynecology  Pharmacology  Nursing and Midwifery | [1.700](http://gateway.isiknowledge.com/gateway/Gateway.cgi?GWVersion=2&SrcAuth=JCR&SrcApp=JCR&DestApp=JCR&KeyRecord=1362-5187) | Q4 | [3.00](https://www.scopus.com/sourceid/24635) | [51](https://www.scimagojr.com/journalsearch.php?q=24635&tip=sid) | 220 | 4 | 9 | 0.87 | Brazil |
| 25 | 2018  (46) | OBSTETRICS & GYNECOLOGY | [ISI](http://mjl.clarivate.com/cgi-bin/jrnlst/jlresults.cgi?PC=MASTER&ISSN=0029-7844), [Scopus](https://www.scopus.com/sourceid/28089), [PubMed](https://www.ncbi.nlm.nih.gov/nlmcatalog/?term=0029-7844), [Embase](https://www.embase.com/search?sb=y&search_query='13625187':is) | \| 5% \| Obstetrics & Gynecology \| \| --- \| --- \| | [7.200](http://gateway.isiknowledge.com/gateway/Gateway.cgi?GWVersion=2&SrcAuth=JCR&SrcApp=JCR&DestApp=JCR&KeyRecord=0029-7844) | Q1 | [9.80](https://www.scopus.com/sourceid/28089) | [245](https://www.scimagojr.com/journalsearch.php?q=28089&tip=sid) | 309 | 24 | 88 | 7.43 | United States |
| 26 | 2019  (47) | Human reproduction | [ISI](http://mjl.clarivate.com/cgi-bin/jrnlst/jlresults.cgi?PC=MASTER&ISSN=0268-1161), [Scopus](https://www.scopus.com/sourceid/27505), [PubMed](https://www.ncbi.nlm.nih.gov/nlmcatalog/?term=0268-1161), [Embase](https://www.embase.com/search?sb=y&search_query='02681161':is) | \| 5% \| Rehabilitation  Physical Therapy & Rehabilitation  Obstetrics & Gynecology \| \| --- \| --- \| | [6.100](http://gateway.isiknowledge.com/gateway/Gateway.cgi?GWVersion=2&SrcAuth=JCR&SrcApp=JCR&DestApp=JCR&KeyRecord=0268-1161) | Q1 | [10.50](https://www.scopus.com/sourceid/27505) | [245](https://www.scimagojr.com/journalsearch.php?q=27505&tip=sid) | 614 | 8 | 7 | 0.48 | Europe and Japan |
| 27 | 2019  (48) | Advances in Integrative Medicine | [ESCI (ISI)](http://mjl.clarivate.com/cgi-bin/jrnlst/jlresults.cgi?PC=MASTER&ISSN=2212-9588),  [Scopus](https://www.scopus.com/sourceid/21100274755),  [Embase](https://www.embase.com/search?sb=y&search_query='22129588':is) | -- | [1.700](http://gateway.isiknowledge.com/gateway/Gateway.cgi?GWVersion=2&SrcAuth=JCR&SrcApp=JCR&DestApp=JCR&KeyRecord=2212-9588) | -- | [2.60](https://www.scopus.com/sourceid/21100274755) | [15](https://www.scimagojr.com/journalsearch.php?q=21100274755&tip=sid) | 247 | 2 | 2 | 0.14 | Australia |
| 28 | 2019  (49) | International Journal of Fertility and Sterility | [ESCI (ISI)](http://mjl.clarivate.com/cgi-bin/jrnlst/jlresults.cgi?PC=MASTER&ISSN=2008-076X), [Scopus](https://www.scopus.com/sourceid/17600155228), [PubMed](https://www.ncbi.nlm.nih.gov/nlmcatalog/?term=2008-076X), [Embase](https://www.embase.com/search?sb=y&search_query='2008076X':is) | Obstetrics & Gynecology | [2.500](http://gateway.isiknowledge.com/gateway/Gateway.cgi?GWVersion=2&SrcAuth=JCR&SrcApp=JCR&DestApp=JCR&KeyRecord=2008-076X) | -- | [4.60](https://www.scopus.com/sourceid/17600155228) | [34](https://www.scimagojr.com/journalsearch.php?q=17600155228&tip=sid) | 296 | 7 | 25 | 1.34 | Iran |
| 29 | 2019  (50) | Journal of Clinical Medicine | [ISI](http://mjl.clarivate.com/cgi-bin/jrnlst/jlresults.cgi?PC=MASTER&ISSN=2077-0383), [PubMed](https://www.ncbi.nlm.nih.gov/nlmcatalog/?term=2077-0383), [Embase](https://www.embase.com/search?sb=y&search_query='13625187':is) | General Medicine | [3.900](http://gateway.isiknowledge.com/gateway/Gateway.cgi?GWVersion=2&SrcAuth=JCR&SrcApp=JCR&DestApp=JCR&KeyRecord=2077-0383) | Q2 | [5.40](https://www.scopus.com/sourceid/21101054449) | [95](https://www.scimagojr.com/journalsearch.php?q=21101054449&tip=sid) | 214 | 8 | 12 | 0.98 | France |
| 30 | 2019  (51) | The Journal of Minimally Invasive Gynecology | [ISI](http://mjl.clarivate.com/cgi-bin/jrnlst/jlresults.cgi?PC=MASTER&ISSN=1553-4650), [Scopus](https://www.scopus.com/sourceid/144915), [PubMed](https://www.ncbi.nlm.nih.gov/nlmcatalog/?term=1553-4650), [Embase](https://www.embase.com/search?sb=y&search_query='13625187':is) | Obstetrics & Gynecology | [4.100](http://gateway.isiknowledge.com/gateway/Gateway.cgi?GWVersion=2&SrcAuth=JCR&SrcApp=JCR&DestApp=JCR&KeyRecord=1553-4650) | Q1 | [4.40](https://www.scopus.com/sourceid/144915) | [88](https://www.scimagojr.com/journalsearch.php?q=144915&tip=sid) | 339 | 5 | 34 | 3.53 | United States |
| 31 | 2020  (52) | Journal of Women's Health | [ISI](http://mjl.clarivate.com/cgi-bin/jrnlst/jlresults.cgi?PC=MASTER&ISSN=1540-9996), [Scopus](https://www.scopus.com/sourceid/19558), [PubMed](https://www.ncbi.nlm.nih.gov/nlmcatalog/?term=1540-9996), [Embase](https://www.embase.com/search?sb=y&search_query='15409996':is) | General Medicine | [3.500](http://gateway.isiknowledge.com/gateway/Gateway.cgi?GWVersion=2&SrcAuth=JCR&SrcApp=JCR&DestApp=JCR&KeyRecord=1540-9996) | Q1 | [5.60](https://www.scopus.com/sourceid/19558) | [107](https://www.scimagojr.com/journalsearch.php?q=19558&tip=sid) | 277 | 6 | 9 | 0.72 | Italy |
| 32 | 2020  (53) | Fertility and Sterility | [ISI](http://mjl.clarivate.com/cgi-bin/jrnlst/jlresults.cgi?PC=MASTER&ISSN=0015-0282), [Scopus](https://www.scopus.com/sourceid/12705), [PubMed](https://www.ncbi.nlm.nih.gov/nlmcatalog/?term=0015-0282), [Embase](https://www.embase.com/search?sb=y&search_query='13625187':is) | \| 5% \| Obstetrics & Gynecology \| \| --- \| --- \| | [6.700](http://gateway.isiknowledge.com/gateway/Gateway.cgi?GWVersion=2&SrcAuth=JCR&SrcApp=JCR&DestApp=JCR&KeyRecord=0015-0282) | Q1 | [10.50](https://www.scopus.com/sourceid/12705) | [230](https://www.scimagojr.com/journalsearch.php?q=12705&tip=sid) | 231 | 9 | 62 | 7.29 | United States and Europe |
| 33 | 2020  (54) | European Journal of Obstetrics & Gynecology and Reproductive Biology | [ISI](http://mjl.clarivate.com/cgi-bin/jrnlst/jlresults.cgi?PC=MASTER&ISSN=0301-2115), [Scopus](https://www.scopus.com/sourceid/12681), [PubMed](https://www.ncbi.nlm.nih.gov/nlmcatalog/?term=0301-2115), [Embase](https://www.embase.com/search?sb=y&search_query='13625187':is) | Obstetrics & Gynecology  Nursing and Midwifery | [2.600](http://gateway.isiknowledge.com/gateway/Gateway.cgi?GWVersion=2&SrcAuth=JCR&SrcApp=JCR&DestApp=JCR&KeyRecord=0301-2115) | Q2 | [4.00](https://www.scopus.com/sourceid/12681) | [111](https://www.scimagojr.com/journalsearch.php?q=12681&tip=sid) | 250 | 5 | 13 | 1.31 | Brazil |
| 34 | 2020  (55) | BMC Women's Health | [ISI](http://mjl.clarivate.com/cgi-bin/jrnlst/jlresults.cgi?PC=MASTER&ISSN=1472-6874), [Scopus](https://www.scopus.com/sourceid/19499), [PubMed](https://www.ncbi.nlm.nih.gov/nlmcatalog/?term=1472-6874), [Embase](https://www.embase.com/search?sb=y&search_query='14726874':is), [DOAJ](https://doaj.org/toc/1472-6874) | General Medicine  Obstetrics & Gynecology | [2.500](http://gateway.isiknowledge.com/gateway/Gateway.cgi?GWVersion=2&SrcAuth=JCR&SrcApp=JCR&DestApp=JCR&KeyRecord=1472-6874) | Q3 | [3.20](https://www.scopus.com/sourceid/19499) | [58](https://www.scimagojr.com/journalsearch.php?q=19499&tip=sid) | 253 | 5 | 4 | 0.74 | Brazil |
| 35 | 2021  (56) | Fertility and Sterility | [ISI](http://mjl.clarivate.com/cgi-bin/jrnlst/jlresults.cgi?PC=MASTER&ISSN=0015-0282), [Scopus](https://www.scopus.com/sourceid/12705), [PubMed](https://www.ncbi.nlm.nih.gov/nlmcatalog/?term=0015-0282), [Embase](https://www.embase.com/search?sb=y&search_query='00150282':is) | \| 5% \| Obstetrics & Gynecology \| \| --- \| --- \| | [6.700](http://gateway.isiknowledge.com/gateway/Gateway.cgi?GWVersion=2&SrcAuth=JCR&SrcApp=JCR&DestApp=JCR&KeyRecord=0015-0282) | Q1 | [10.50](https://www.scopus.com/sourceid/12705) | [230](https://www.scimagojr.com/journalsearch.php?q=12705&tip=sid) | 243 | 6 | 45 | 5.67 | Japan |
| 36 | 2022  (57) | Journal of Obstetrics and Gynaecology | [ISI](http://mjl.clarivate.com/cgi-bin/jrnlst/jlresults.cgi?PC=MASTER&ISSN=0144-3615), [Scopus](https://www.scopus.com/sourceid/27604), [PubMed](https://www.ncbi.nlm.nih.gov/nlmcatalog/?term=0144-3615), [Embase](https://www.embase.com/search?sb=y&search_query=%2701443615%27:is) | Obstetrics and Gynaecology | 1.3 | Q4 | [1.80](https://www.scopus.com/sourceid/27604) | 55 | 149 | 8 | 0 | - | Iran |
| 37 | 2021  (58) | [Fertility and Sterility](http://www.fertstert.org/) | [ISI](http://mjl.clarivate.com/cgi-bin/jrnlst/jlresults.cgi?PC=MASTER&ISSN=0015-0282), [Scopus](https://www.scopus.com/sourceid/12705), [PubMed](https://www.ncbi.nlm.nih.gov/nlmcatalog/?term=0015-0282), [Embase](https://www.embase.com/search?sb=y&search_query=%2700150282%27:is) | 5% Obstetrics and Gynaecology | [6.700](http://gateway.isiknowledge.com/gateway/Gateway.cgi?GWVersion=2&SrcAuth=JCR&SrcApp=JCR&DestApp=JCR&KeyRecord=0015-0282) | Q1 | [10.50](https://www.scopus.com/sourceid/12705) | [230](https://www.scimagojr.com/journalsearch.php?q=12705&tip=sid) | 243 | 6 | 7 | 1.40 | United States |
| 38 | 2022  (59) | European Journal of Obstetrics & Gynecology and Reproductive Biology | [ISI](http://mjl.clarivate.com/cgi-bin/jrnlst/jlresults.cgi?PC=MASTER&ISSN=0301-2115), [Scopus](https://www.scopus.com/sourceid/12681), [PubMed](https://www.ncbi.nlm.nih.gov/nlmcatalog/?term=0301-2115), [Embase](https://www.embase.com/search?sb=y&search_query=%2703012115%27:is) | Obstetrics and Gynaecology | [2.600](http://gateway.isiknowledge.com/gateway/Gateway.cgi?GWVersion=2&SrcAuth=JCR&SrcApp=JCR&DestApp=JCR&KeyRecord=0301-2115) | Q2 | [4.00](https://www.scopus.com/sourceid/12681) | [111](https://www.scimagojr.com/journalsearch.php?q=12681&tip=sid) | 302 | 6 | 4 | 2.15 | Lebanon |
| 39 | 2022  (60) | [Fertility and Sterility](http://www.fertstert.org/) | [ISI](http://mjl.clarivate.com/cgi-bin/jrnlst/jlresults.cgi?PC=MASTER&ISSN=0015-0282), [Scopus](https://www.scopus.com/sourceid/12705), [PubMed](https://www.ncbi.nlm.nih.gov/nlmcatalog/?term=0015-0282), [Embase](https://www.embase.com/search?sb=y&search_query=%2700150282%27:is) | 5% Obstetrics and Gynaecology | [6.700](http://gateway.isiknowledge.com/gateway/Gateway.cgi?GWVersion=2&SrcAuth=JCR&SrcApp=JCR&DestApp=JCR&KeyRecord=0015-0282) | Q1 | [10.50](https://www.scopus.com/sourceid/12705) | [230](https://www.scimagojr.com/journalsearch.php?q=12705&tip=sid) | 234 | 6 | 16 | 6.39 | Japan |
| 40 | 2023  (61) | [Journal of Clinical Medicine](http://www.mdpi.com/journal/jcm) | [ISI](http://mjl.clarivate.com/cgi-bin/jrnlst/jlresults.cgi?PC=MASTER&ISSN=0015-0282), [Scopus](https://www.scopus.com/sourceid/12705), [PubMed](https://www.ncbi.nlm.nih.gov/nlmcatalog/?term=0015-0282), [Embase](https://www.embase.com/search?sb=y&search_query=%2700150282%27:is) | General Medicine | [3.900](http://gateway.isiknowledge.com/gateway/Gateway.cgi?GWVersion=2&SrcAuth=JCR&SrcApp=JCR&DestApp=JCR&KeyRecord=2077-0383) | Q2 | [5.40](https://www.scopus.com/sourceid/21101054449) | [95](https://www.scimagojr.com/journalsearch.php?q=21101054449&tip=sid) | 224 | 7 | 1 | 1.72 | Germany |
| 41 | 2023  (62) | [Fertility and Sterility](http://www.fertstert.org/) | [ISI](http://mjl.clarivate.com/cgi-bin/jrnlst/jlresults.cgi?PC=MASTER&ISSN=0015-0282), [Scopus](https://www.scopus.com/sourceid/12705), [PubMed](https://www.ncbi.nlm.nih.gov/nlmcatalog/?term=0015-0282), [Embase](https://www.embase.com/search?sb=y&search_query=%2700150282%27:is) | 5% Obstetrics and Gynaecology | [6.700](http://gateway.isiknowledge.com/gateway/Gateway.cgi?GWVersion=2&SrcAuth=JCR&SrcApp=JCR&DestApp=JCR&KeyRecord=0015-0282) | Q1 | [10.50](https://www.scopus.com/sourceid/12705) | [230](https://www.scimagojr.com/journalsearch.php?q=12705&tip=sid) | 298 | 9 | 1 | 1.60 | China |
| 42 | 2022  (63) | Scientific Reports | [ISI](http://mjl.clarivate.com/cgi-bin/jrnlst/jlresults.cgi?PC=MASTER&ISSN=0015-0282), [Scopus](https://www.scopus.com/sourceid/12705), [PubMed](https://www.ncbi.nlm.nih.gov/nlmcatalog/?term=0015-0282), [Embase](https://www.embase.com/search?sb=y&search_query=%2700150282%27:is) | General Medicine | [4.600](http://gateway.isiknowledge.com/gateway/Gateway.cgi?GWVersion=2&SrcAuth=JCR&SrcApp=JCR&DestApp=JCR&KeyRecord=2045-2322) | Q2 | [7.50](https://www.scopus.com/sourceid/21100200805) | [282](https://www.scimagojr.com/journalsearch.php?q=21100200805&tip=sid) | 146 | 4 | 3 | 0.50 | Iran |
| 43 | 2023  (64) | [Journal of Clinical Medicine](http://www.mdpi.com/journal/jcm) | [ISI](http://mjl.clarivate.com/cgi-bin/jrnlst/jlresults.cgi?PC=MASTER&ISSN=0015-0282), [Scopus](https://www.scopus.com/sourceid/12705), [PubMed](https://www.ncbi.nlm.nih.gov/nlmcatalog/?term=0015-0282), [Embase](https://www.embase.com/search?sb=y&search_query=%2700150282%27:is) | General Medicine | [3.900](http://gateway.isiknowledge.com/gateway/Gateway.cgi?GWVersion=2&SrcAuth=JCR&SrcApp=JCR&DestApp=JCR&KeyRecord=2077-0383) | Q2 | [5.40](https://www.scopus.com/sourceid/21101054449) | [95](https://www.scimagojr.com/journalsearch.php?q=21101054449&tip=sid) | 215 | 7 | 0 | - | Spain |
| 44 | 2023  (65) | Archives of Gynecology and Obstetrics | [ISI](http://mjl.clarivate.com/cgi-bin/jrnlst/jlresults.cgi?PC=MASTER&ISSN=0015-0282), [Scopus](https://www.scopus.com/sourceid/12705), [PubMed](https://www.ncbi.nlm.nih.gov/nlmcatalog/?term=0015-0282), [Embase](https://www.embase.com/search?sb=y&search_query=%2700150282%27:is) | General Medicine  Obstetrics and Gynaecology | [2.600](http://gateway.isiknowledge.com/gateway/Gateway.cgi?GWVersion=2&SrcAuth=JCR&SrcApp=JCR&DestApp=JCR&KeyRecord=0932-0067) | Q3 | [4.50](https://www.scopus.com/sourceid/30082) | [79](https://www.scimagojr.com/journalsearch.php?q=30082&tip=sid) | 245 | 5 | 0 | - | Iran |
| 45 | 2022  (66) | The Lancet | [ISI](http://mjl.clarivate.com/cgi-bin/jrnlst/jlresults.cgi?PC=MASTER&ISSN=0140-6736), [Scopus](https://www.scopus.com/sourceid/16590), [PubMed](https://www.ncbi.nlm.nih.gov/nlmcatalog/?term=0140-6736), [Embase](https://www.embase.com/search?sb=y&search_query=%2701406736%27:is) | \| 1% \| General Medicine \| \| --- \| --- \| | [168.900](http://gateway.isiknowledge.com/gateway/Gateway.cgi?GWVersion=2&SrcAuth=JCR&SrcApp=JCR&DestApp=JCR&KeyRecord=0140-6736) | Q1 | [133.20](https://www.scopus.com/sourceid/16590) | [855](https://www.scimagojr.com/journalsearch.php?q=16590&tip=sid) | 734 | 14 | 42 | 15.47 | Africa  Australia  Europe  North America  South America |
| 46 | 2021  (67) | BMC Women's Health | [ISI](http://mjl.clarivate.com/cgi-bin/jrnlst/jlresults.cgi?PC=MASTER&ISSN=1472-6874), [Scopus](https://www.scopus.com/sourceid/19499), [PubMed](https://www.ncbi.nlm.nih.gov/nlmcatalog/?term=1472-6874), [Embase](https://www.embase.com/search?sb=y&search_query=%2714726874%27:is), [DOAJ](https://doaj.org/toc/1472-6874) | Obstetrics and Gynaecology | [2.500](http://gateway.isiknowledge.com/gateway/Gateway.cgi?GWVersion=2&SrcAuth=JCR&SrcApp=JCR&DestApp=JCR&KeyRecord=1472-6874) | Q3 | [3.20](https://www.scopus.com/sourceid/19499) | [58](https://www.scimagojr.com/journalsearch.php?q=19499&tip=sid) | 325 | 6 | 13 | 2.40 | Japan |
| 47 | 2023  (68) | Journal of Nursing and Midwifery Sciences | [ESCI (ISI)](http://mjl.clarivate.com/cgi-bin/jrnlst/jlresults.cgi?PC=MASTER&ISSN=2345-5756), [Scopus](https://www.scopus.com/sourceid/21101041956) | General Nursing | [0.700](http://gateway.isiknowledge.com/gateway/Gateway.cgi?GWVersion=2&SrcAuth=JCR&SrcApp=JCR&DestApp=JCR&KeyRecord=2345-5756) |  | [0.60](https://www.scopus.com/sourceid/21101041956) | [3](https://www.scimagojr.com/journalsearch.php?q=21101041956&tip=sid) | 329 | 6 | 0 | - | Iran |
