# Supplementary material for: The reporting quality and spin of randomized controlled trials of endometriosis pain: Methodological study based on CONSORT extension on abstracts
Source: PLoS One. 2024 May 2;19(5):e0302108. doi: 10.1371/journal.pone.0302108 (PMC11065215; doi:10.1371/journal.pone.0302108)
Supplement: Attachment — Submitted filename: Plos Review.docx [file pone.0302108.s004.docx]

The authors analyzed the adherence to CONSORT for abstracts in 35 abstracts on RCT publications about endometriosis pelvic pain published between 2010 and 2021. They found that a mean of 8.54 from the 16 CONSORT-A was reported showing that there was poor overall adherence to CONSORT-A in these abstracts. In addition to that, they analyzed if studies with a non-significant primary endpoint misinterpreted this or if they reported secondary endpoints that were significant instead. They found that about 67% of these 18 abstracts had such a bias in the interpretation of results.

Even though the topic is important and the manuscript has some strengths, some points have been noticed while reading that should definitely be revised and adapted.

Flowchart:

The Flowchart that is shown in figure 1 should be improved. In the first 3 steps the reasons for exclusion of RCT-abstracts are not reported. This is necessary for the reader to understand the process of data collection and should be added. In addition to that the final number of abstracts for analysis is 35. This is correct for the analysis of adherence to CONSORT-A but as the authors write in their methods section they only included abstracts with non-significant primary endpoints (n = 18?). This should be added to the Flowchart to avoid confusion.

Abstract:

- In the abstract and title the word spin is used for the sort of interpretation bias analyzed in the paper. This should be defined in the abstract as the meaning of spin might not be clear for every reader.
- It is said that 35 abstracts are included but this wrong for the analysis of spin. Additionally the number of abstracts initially found should be added.
- It should be defined if only the reporting of the CONSORT-A items is checked or if the quality/ completeness is also checked. If yes: How?

Introduction

- It should be added that abstracts are not only the source of knowledge in countries with low income but also the decision maker if a fulltext publication is worth buying.

Methods

- Please explain why you need information from fulltext articles if abstracts are of interest
- Why do you chose a time of 2 years after publication of CONSORT-A? Why would 1 year not have been enough time?
- Why are significant primary endpoints excluded from spin analysis? Is spin really absolutely impossible there?
- open access yes/ no should be included as a characteristic and open access and non open access abstracts should be compared
- Please define when a CONSORT-A item was reported adequately
- Is the overall reporting score the sum of items? Please define it as it is the primary endpoint of the study. This should be changed in the section about the primary endpoint as this is used to identify the quality
- To the 3 point in spins in the results section: how can this be checked when abstracts with multiple primary endpoints are excluded?
- The last sentence in the “Spins in conclusions sections” should be part of the exclusion criteria
- are all points according to spin of the same importance or is there a weighting possible/ necessary?

Statistical analysis:

- Only the primary endpoint/comparison should have a hypothesis testing? → why are there so many tests planned?
- In order to find associations and influencing factors on reporting quality a regression model (for example poisson regression) should be performed instead of the testing that is used. The authors should fit to regression models (one for CONSORT-A items and one for spin) so that a better (and adjusted) interpretation of the factors is possible. I ask the authors to add to these regression analyses.

Results

- The adherence to CONSORT-A should be described in more detail as it is the primary endpoint of the study.
- Please delete testing and add the results of regression modeling

Discussion

- When you analyze factors that affect the quality then this should be done with regression models
- General question: Did you check if the item was reported or was the quality and completeness of the reporting checked as well?
- “none of the abstracts presented precise data as required” what do you mean with this statement?
- “most items identified that were suboptimal” it was not reported at all or insufficiently
- why are allocation concealment and blinding the most important points?
- Please add the information on the medical field that was investigated in reference 17 and 18
- Why was information on endpoints extracted from the full texts when abstracts were reviewed?
- I could not find the results on IF that are mentioned in the discussion
- The study registration is mentioned as a method to improve quality. But how is ensured that these are complete and correct?

Conclusion:

The study only shows the need for improvement of RCT abstracts but not of RCT reports. Please change the conclusion accordingly.

The own results and the interpretation should be discussed in more detail

The authors should revise the language to improve readability in some parts of the manuscript.
