# Supplementary material for: The reporting quality and spin of randomized controlled trials of endometriosis pain: Methodological study based on CONSORT extension on abstracts
Source: PLoS One. 2024 May 2;19(5):e0302108. doi: 10.1371/journal.pone.0302108 (PMC11065215; doi:10.1371/journal.pone.0302108)
Supplement: Attachment — Submitted filename: Plos Review- Final response2.docx [file pone.0302108.s005.docx]

We highly appreciate the reviewers' and editors’ insightful and helpful comments on our manuscript entitled “**The reporting quality and spin of randomized controlled trials of endometriosis pain: Methodological study based on CONSORT extension on abstracts**”. We appreciate the opportunity to address the reviewer's comments. Extending our search to include articles published until **December 2023** was indeed a challenging and time-consuming process. Despite the difficulty, we were able to identify and **include 12 additional papers**, which have been thoroughly analyzed and integrated into the revised manuscript. We believe that these updates have significantly enhanced the comprehensiveness and robustness of our findings., and we are confident that these revisions will strengthen the overall contribution of our study to the field.

**Flowchart:**

*The Flowchart that is shown in figure 1 should be improved. In the first 3 steps the reasons for exclusion of RCT-abstracts are not reported. This is necessary for the reader to understand the process of data collection and should be added. In addition to that the final number of abstracts for analysis is 35. This is correct for the analysis of adherence to CONSORT-A but as the authors write in their methods section they only included abstracts with non-significant primary endpoints (n = 18?). This should be added to the Flowchart to avoid confusion.*

**Response:**

The flowchart now incorporates the rationales for excluding RCT abstracts. Additionally, the data collection procedure has been incorporated and emphasized. Out of the initially identified 47 eligible abstracts, all were ultimately included in the final analysis. However, during the spin assessment, it was observed that only 21 papers (in new search)exhibited non-significant primary endpoints. To enhance transparency, a dedicated step for spin analysis has been introduced to the flowchart.

**Abstract:**

- *In the abstract and title the word spin is used for the sort of interpretation bias analyzed in the paper. This should be defined in the abstract as the meaning of spin might not be clear for every reader.*

**Response:** We added the definition of spin to the abstract and highlighted.

- *It is said that 35 abstracts are included but this wrong for the analysis of spin. Additionally the number of abstracts initially found should be added.*

**Response:** We conducted a two-phase analysis of the included abstracts. Initially, we scrutinized the adherence to CONSORT guidelines in 47 eligible abstracts. Subsequently, all incorporated papers underwent an assessment for spin, revealing that only 21 papers featured non-significant primary endpoints.

- *It should be defined if only the reporting of the CONSORT-A items is checked or if the quality/ completeness is also checked. If yes: How?*

**Response:** Our study's main aim was to evaluate the reporting quality of RCT abstracts, focusing on CONSORT-A criteria.

**Introduction**

- *It should be added that abstracts are not only the source of knowledge in countries with low income but* *also the decision maker if a fulltext publication is worth buying.*

**Response:** In response to the reviewer's suggestions, the document was revised and highlighted.

**Methods**

- *Please explain why you need information from fulltext articles if abstracts are of interest.*

**Response:** To assess both primary and secondary outcomes during spin analysis, a thorough examination of the full-text articles is necessary. The primary outcome should be explicitly stated in either the abstract or the full text. In cases where the primary outcome is not clearly defined, we relied on the outcome declared in the sample size calculations as the primary measure. If such information was not available, we inferred the primary outcome solely from the stated objectives of the study.

- *Why do you chose a time of 2 years after publication of CONSORT-A? Why would 1 year not have been enough time?*

**Response:**This timeframe allows ample opportunity for researchers to implement CONSORT for abstracts effectively. Additionally, it accounts for the typical duration encompassing peer review processes and the final publication in journals. This extended period ensures a comprehensive evaluation of the integration and adoption of CONSORT-A guidelines within the scholarly community.

- *Why are significant primary endpoints excluded from spin analysis? Is spin really absolutely impossible there?*

**Response:** As the definition of the spin, it may occurs when the between groups effect were non-significant. The exclusion of significant primary endpoints from the spin analysis was not due to the belief that spin is absolutely impossible in those cases. Rather, our decision was based on the methodological focus of our study, which specifically aimed to assess the reporting quality and spin of randomized controlled trials of endometriosis pain. As such, our analysis was centered on the reporting of outcomes and the potential influence of spin on the interpretation of trial results, rather than the presence or absence of statistically significant findings.Therefore, the articles that had significant primary endpoints excluded from spin analysis.

- *open access yes/ no should be included as a characteristic and open access and non open access abstracts should be compared*

**Response:** based on reviewer suggestion it was added and highlighted.

- *Please define when a CONSORT-A item was reported adequately*

**Response:** A CONSORT-A item was considered reported adequately when the abstract provided clear and complete information in accordance with the specific guideline outlined by CONSORT for abstracts. This entails fulfilling the criteria specified for each item, ensuring a comprehensive and transparent presentation of essential study details within the limitations of the abstract format.

- *Is the overall reporting score the sum of items? Please define it as it is the primary endpoint of the study. This should be changed in the section about the primary endpoint as this is used to identify the quality*.

**Response:** Yes, the overall reporting score is derived from the sum of items assessed in accordance with CONSORT-A guidelines. It serves as the primary endpoint of the study, indicating the quality of reporting in RCT abstracts. Based on reviewer sugestion it was added.

- *To the 3 point in spins in the results section: how can this be checked when abstracts with multiple primary endpoints are excluded?*

**Response:** We have thoroughly reviewed the spin analysis and have identified a few discrepancies that we would like to address. First, we acknowledge that we omitted the sentence "more than one primary outcome" in the spin evaluation methods section. This was an oversight on our part, and we apologize for any confusion it may have caused.

Second, we have re-assessed all of the excluded papers that had multiple primary outcomes. Some papers had multiple primary outcomes, but none of these were used in the spin analysis because the main outcomes had significant results. The titles, primary outcomes, and reasons for excluding these studies are as follows:

- Postoperative administration of dienogest plus estradiol valerate versus levonorgestrel-releasing intrauterine device for prevention of pain relapse and disease recurrence in endometriosis patients (**Primary outcomes**: pain and disease recurrence rate) significant result
- Treatment of Endometriosis-Associated Pain with Elagolix, an Oral GnRH Antagonist **(Primary outcomes**: non menstrual pelvic-pain and dysmenorrhea). significant result
- Beneficial effects of oral lactobacillus on pain severity in women suffering from endometriosis: A pilot placebo-controlled randomized clinical trial (**Primary outcomes:** pain severity using (VAS) scores for dysmenorrhea, dyspareunia and chronic pelvic pain) significant result
- Effectiveness of the association micronized N-Palmitoylethanolamine (PEA)-transpolydatin in the treatment of chronic pelvic pain related to endometriosis after laparoscopic assessment: A pilot study (**Primary outcomes:** pelvic pain, dysmenorrhea and dyspareunia). significant result
- Letrozole and norethisterone acetate versus letrozole and triptorelin in the treatment of endometriosis related pain symptoms: a randomized controlled trial **(Primary outcomes**: pain symptoms( significant result
- Maintenance therapy with dienogest following gonadotropin-releasing hormone agonist treatment for endometriosis-associated pelvic pain (**Primary outcomes:** pelvic pain and irregular uterine bleeding) significant result
- Effect of abdominal acupuncture on pain of pelvic cavity in patients with endometriosis **(primary outcome:** the scores of McGill pain questionaire, level of serum CA125, average value of the radial line of endometrial cyst of ovary and the sum of 3 radial lines of the uterus of patients with adenomyosis as the complication) significant result
- Antioxidant supplementation reduces endometriosis-related pelvic pain in humans(**primary outcomes:** peritoneal fluid biomarkers for oxidation or affect pelvic pain) significant result
- Repetitive transcranial magnetic stimulation therapy (Rtms) for endometriosis patients with refractory pelvic chronic pain: A pilot study (**primary outcomes:** tolerance, pain change and Quality of life) significant result
- Does Nomegestrol Acetate plus 17β-Estradiol Oral Contraceptive Improve Endometriosis-Associated Chronic Pelvic Pain in Women? (**primary outcomes:** pain symptoms, Quality of life, Female Sexual Function Index, and Female Sexual Distress Scale) significant result
- Treatment of endometriosis-associated pain with linzagolix, an oral gonadotropin-releasing hormone–antagonist: a randomized clinical trial )primary outcomes: number of responders (R30% reduction in overall pelvic pain) after 12 weeks. Other endpoints included dysmenorrhea, non-menstrual pelvic pain, serum estradiol, amenorrhea, quality of life (QoL) measures, and bone mineral density (BMD)).
- *The last sentence in the “Spins in conclusions sections” should be part of the exclusion criteria*

**Response:** We had two groups of eligibility criteria, one for quality assessment and the other for spin assessment. The above sentence is the exclusion for spin and concordance with the previous comment we omitted it.

- *Are all points according to spin of the same importance or is there a weighting possible/ necessary*?

**Response:** It was the same.

**Statistical analysis:**

- *Only the primary endpoint/comparison should have a hypothesis testing? → why are there so many tests planned?*

**Response:** All of the tests were performed based on the primary endpoints

- *In order to find associations and influencing factors on reporting quality a regression model (for example poisson regression) should be performed instead of the testing that is used. The authors should fit to regression models (one for CONSORT-A items and one for spin) so that a better (and adjusted) interpretation of the factors is possible. I ask the authors to add to these regression analyses.*

**Response:** we encountered challenges in conducting regression analysis due to the limited sample size of 47 included articles. The small sample size resulted in a low power, making it difficult to draw statistically significant conclusions from the regression models. In accordance with reviewer’s comment we performed it. The result is as follow:

we utilized a multivariable linear regression with the backward method to assess the factors influencing the Quality of Reporting (QoR). We considered the QoR as the dependent variable and included the word count of the abstract, the indexing information of the journal (such as whether it is indexed in PubMed, Scopus, and Embase), and journal metrics (including impact factor (IF), cite score, and H-index) as the independent factors.As seen in table 3, with increasing one unit in word count and also the cite score, the mean change in QoR is increased (beta for word count=0.017, -value<0.001 and beta for cite score=0.688, p-value<0.001).

**Results**

- *The adherence to CONSORT-A should be described in more detail as it is the primary endpoint of the study*

Response: It was done so.

- *Please delete testing and add the results of regression modeling*

Response: We provided a detailed explanation in our previous comments. we utilized a multivariable linear regression with the backward method to assess the factors influencing the Quality of Reporting (QoR).

**Discussion**

- *When you analyze factors that affect the quality then this should be done with regression models.*

Response: Although only 47 articles were included in the analysis, we conducted regression analysis despite the small sample size to comply with the reviewer's suggestion. However, the power of the analysis was compromised due to the limited sample size

- *General question: Did you check if the item was reported or was the quality and completeness of the reporting checked as well?*

Response: We did not only check if the items were reported but also evaluated the quality and completeness of the reporting using the CONSORT-A extension. The CONSORT-A extension includes 11 related sub-items that assess the clarity, accuracy, and consistency of the reported information.

- *none of the abstracts presented precise data as required” what do you mean with this statement?*

Response: **i**n the statement "none of the abstracts presented precise data as required," we meant to convey that the abstracts of the included RCTs did not adequately provide specific numerical data as required by the CONSORT-A guidelines.

- *“most items identified that were suboptimal” it was not reported at all or insufficiently*

Response: it was added to the result section (as follow) and also it was stated in table2

Furthermore, evaluating each item of CONSORT-A checklist, in titles of 16 (45.7%) papers, identified the study as randomized. In most of abstracts the eligibility criteria for participants was described. *Also, in 30 (85.7%) of abstracts the study design was stated in title or in the method* *section,* even *though randomization part is a very important, only about half of the articles reported it generally and about 17.14% of them reported it in detail.* The detail of CONSORT items is shown in table 2.“

- *why are allocation concealment and blinding the most important points?*

Response: These two points were the most important to certify the authenticity of results. to identify the selection bias, it is important to indicate how participants were allocated and randomized to the intervention. Understanding this information allows readers to determine whether the findings of the trial are biased in favor of the new therapy

- *Please add the information on the medical field that was investigated in reference 17 and 18*

Response: It was added and highlighted.

- *Why was information on endpoints extracted from the full texts when abstracts were reviewed?*

**Response:** to differentiate the primary outcome/outcomes from secondary outcomes, somtimes it was needed to extract the endpoints from the fulltext.

- *I could not find the results on IF that are mentioned in the discussion*.

**Response:** It was added.

- *The study registration is mentioned as a method to improve quality. But how is ensured that these are complete and correct?*

**Response:** it was out of the scope and objectives of our study. We just reported it in table 2.

**Conclusion:**

- *The study only shows the need for improvement of RCT abstracts but not of RCT reports. Please change the conclusion accordingly.*

**Response**: It was revised.

*The authors should revise the language to improve readability in some parts of the manuscript.*

**Response:** It was done

- *The study is merthodologically sound and well presented, however, I have one major concern which is the period of study for article selection. The authors indicate that the articles published until Sept 2021 has been included. It is possible that the authors might have missed some crucial data on the trials published in the past 2 years. Hence I suggest the authors to update the search by including articles published until September 2023 and present the updated results.*

**Response:** Extending our search to include articles published until December 2023 was indeed a challenging and time-consuming process. Despite the difficulty, we were able to identify and **include 12 additional papers**, which have been thoroughly analyzed and integrated into the revised manuscript. We believe that these updates have significantly enhanced the comprehensiveness and robustness of our findings
