# Supplementary material for: The reporting quality and spin of randomized controlled trials of endometriosis pain: Methodological study based on CONSORT extension on abstracts
Source: PLoS One. 2024 May 2;19(5):e0302108. doi: 10.1371/journal.pone.0302108 (PMC11065215; doi:10.1371/journal.pone.0302108)
Supplement: Attachment — Submitted filename: response to Reviewer2.docx [file pone.0302108.s006.docx]

Reviewer #2: Introduction:
Aim (ii) should be answered using a regression model.

**Response:** we performed regression analysis and the result were added.

Assessment of reporting quality:
Examples in a supplementary file that show examples for adequately AND insufficient reporting. This would help the reader to understand data collection

Main outcome measure is not adequately defined: It is called quality. Please define it more (as a score with the range of possible values).

Statistical Analysis:
The Analysis should be performed in a correct and adequate way. Currently testing is performed without taking into account the problem of multiple testing. (The level of significance and if it was corrected due to multiple testing must be explained)

**Response:** Wherever we compare a parameter in multiple subgroups we adjust the level of significance. For example, when we want to compare QoR in subgroups of IF quartiles we used Bonferroni post hoc test to taking into account the problem of multiple testing. We added it in the method section.

Please do not use the word “incidence” when describing figure 3

**Response:** we corrected and changed it to spin intensity.

An insufficient linear regression is performed.
Regression analysis should be the main method to answer the question of factors associated with quality of abstract-reporting.
1) More factors should be included in the analysis (all that are interesting and currently used in hypothesis testing)
◊ Word count, FWCI, year, gynecological journal (yes/ no), open access (yes/no), word count, impact factor, cite score
⎝ Please perform regression without variable selection and choose all the variables that are currently analyzed with tests

**Response:** In regression analysis we need more sample size. “the ratio of the sample size to the number of parameters in a regression model should be at least 20 to 1.” (<https://doi.org/10.1016/j.aucc.2012.07.002>) so we couldn’t perform multivariate regression analysis. But in accordance to the reviewer’s comment, we performed it and added to the result section.

Assessing the factors influencing the QoR in the abstracts of RCTs is crucial for understanding the potential determinants of reporting quality in scientific literature. we employed a multivariable linear regression model to investigate the impact of various independent variables on the QoR of RCT abstracts focusing on endometriosis pelvic pain. The independent variables considered in our analysis included the word count of the abstract, indexing in databases such as PubMed, Scopus, and ISI, Field-Weighted Citation Impact (FWCI), publication in a gynecological journal, open access status, Impact Factor (IF), Cite Score, and H-index.

2) Analysis of spin (yes/ no) must be conducted with logistic regression and not with the currently performed testing.

**Response:** As in regression analysis we need at least 20 sample sizes per each independent variable, we couldn’t perform multivariate logistic regression. So, we performed univariable logistic regression. The result is added.

to evaluate the factors influencing spin in the reporting of research findings, logistic regression analysis was conducted. The independent variables considered in the analysis included the word count of the abstract, indexing status in PubMed (yes/no), indexing status in Scopus (yes/no), indexing status in ISI (yes/no), FWCI, designation as a gynecological journal (yes/no), publication in an open-access journal (yes/no), IF, Cite Score, and H-index. These variables were entered into the univariable linear regression model to assess their individual relationships with the presence of spin. However, the analysis did not reveal any statistically significant relationships between the independent variables and the presence of spin in the abstracts. Additionally, the model encountered convergence issues specifically for the variables related to indexing in PubMed, Scopus, and ISI, indicating potential limitations in the modeling approach for these factors
