# Supplementary material for: The reporting quality and spin of randomized controlled trials of endometriosis pain: Methodological study based on CONSORT extension on abstracts
Source: PLoS One. 2024 May 2;19(5):e0302108. doi: 10.1371/journal.pone.0302108 (PMC11065215; doi:10.1371/journal.pone.0302108)
Supplement: Attachment — Submitted filename: Response 3 to reviewer 2.docx [file pone.0302108.s007.docx]

**Dear reviewer 2**

We have carefully considered and incorporated your feedback into our manuscript for the third time. We hope that the revisions made align with your expectations and are deemed acceptable. Thank you for your continued guidance and support throughout this process.

**1-Reviewer #2:**” *Incomplete sentence “…Also, the decision maker if a full-text publication is worth buying These factors indicate* …

-Response: Based on the reviewer's comments, the revised paragraph was added.

These inferences are likely higher among physicians in low- and middle-income countries due to the unavailability of full-text publications. Similarly, patients and the public do not always have access to journal articles. Furthermore, determining whether a full-text publication is worth purchasing is crucial. These factors highlight the importance of reporting quality, clarity, and accuracy during the initial evaluation of RCT abstracts.

2*-Logistic Regression is mentioned in the abstract and the results section. It should also be part of the methods.*

-Response: Based on the reviewer's comments, it was added to the method section.

3-*Change “Furthermore, we found that most articles were single center (n=30, 63.8%)” to
„**Furthermore, we found that most articles described single center (n=30, 63.8%) studies*”

-Response: Based on the reviewer's suggestion, it was changed and highlighted.

4-*Page 19 in the results section words like “interesting” should be avoided*.

-Response: Based on the reviewer's comment, the revised paragraph was added and highlighted.

Our findings revealed valuable insights into the relationship between these independent variables and the QoR of abstracts

*5-Table 3: please explain in methods what Standardized coeffcients means.*

-Response: Based on the reviewer's comment, the revised paragraph was added and highlighted.

Furthermore, to evaluate the strength and direction of the relationships between these independent variables and the QoR, we employed standardized coefficients, also known as Beta coefficients, in our multivariable linear regression model. Beta coefficients, or standardized coefficients, serve as a method to assess the magnitude and orientation of the association among variables that are denoted in disparate units or scales. The process of standardizing these coefficients normalizes the variables to a uniform scale, thereby simplifying the process of comparison

6-*Please explain how the Bias-corrected and accelerated (Bca) 95% confidence interval was computed. This is unclear as the explanation is missing.*

-Response: BCA method allows for the implementation of three corrections to the standard confidence interval. These corrections account for nonnormality, the bias and the nonconstant standard error of the bootstrap distribution, which is often not symmetric in the case of risk measures
